# Neurosurgical treatment and outcome patterns of meningioma in Sweden: a nationwide registry-based study

**DOI:** 10.1007/s00701-019-03799-3

**Published:** 2019-01-24

**Authors:** Alba Corell, Erik Thurin, Thomas Skoglund, Dan Farahmand, Roger Henriksson, Bertil Rydenhag, Sasha Gulati, Jiri Bartek, Asgeir Store Jakola

**Affiliations:** 1 Department of Neurosurgery, Sahlgrenska University Hospital, Gothenburg, Sweden; 2 Institute of Neuroscience and Physiology, Sahlgrenska Academy, University of Gothenburg, Gothenburg, Sweden; 3 Department of Neurology, Sahlgrenska University Hospital, Blå stråket 5, 41345 Gothenburg, Sweden; 4 Regional Cancer Centre Stockholm, Stockholm, Gotland Sweden; 5 Department of Radiation Science and Oncology, University hospital, Umeå, Sweden; 6 Department of Neurosurgery, St. Olavs University Hospital, Trondheim, Norway; 7 Department of Neuromedicine and Movement Science, Norwegian University of Science and Technology, Trondheim, Norway; 8 Department of Neurosurgery, Karolinska University Hospital, Stockholm, Sweden; 9 Department of Clinical Neuroscience and Department of Medicine, Karolinska Institutet, Stockholm, Sweden; 10 Department of Neurosurgery, Copenhagen University Hospital Rigshospitalet, Copenhagen, Denmark

**Keywords:** Neurosurgery, Meningioma, Health registry, Population-based

## Abstract

**Background:**

Surgery is the main treatment modality for intracranial meningiomas, but data on short-term surgical outcome are limited. The aim of this Swedish nationwide registry-based study was to benchmark the 30-day complication rate in a cohort of meningioma patients using data from the Swedish brain tumor registry (SBTR). Furthermore, we investigated outcomes for asymptomatic patients.

**Methods:**

Data were collected from the SBTR for all adults with histopathologically verified intracranial meningioma between 2009 and 2015. Patient symptoms, tumor characteristics, and complications within 30 days postoperatively were analyzed.

**Results:**

In total, 2324 patients, with a mean age of 58.7 years (SD 13.5), underwent surgery for intracranial meningioma and 14.1% of the patients were asymptomatic before the intervention. The most common symptom prior to treatment was focal deficit, which occurred in 1450 patients (62.4%). Moreover, within 30 days after surgery, 344 patients (14.8%) developed new neurological deficits and new-onset seizures occurred in 105 patients (4.5%), while 8.3% of asymptomatic patients developed neurological deficit and 3.7% new-onset seizures. Due to complications, reoperations were performed in 120 patients (5.2%). The postoperative 30-day mortality in the whole cohort was 1.5%.

**Conclusion:**

This study benchmarks the 30-day complication rate after meningioma surgery and provides outcome data in the highly relevant group of asymptomatic patients using data from the Swedish brain tumor registry. Since surgical decision-making is a careful consideration of short-term risk versus long-term benefit, this information may be useful for both caregivers and patients.

## Introduction

Meningiomas are the most common intracranial extra-axial tumor and most of them are benign (i.e., World Health Organization (WHO) grade I) [6, 24, 30]. Common symptoms include seizures, headache, personality change, confusion, and focal deficit depending on anatomical location [33]. However, meningiomas may also be an incidental finding in asymptomatic patients [42]. Increased availability of MRI has led to more incidental findings of benign intracranial tumors [1, 37], and referral of asymptomatic patients with small incidental meningiomas is frequently encountered in clinical practice. Meningiomas usually have a slow growth rate, and literature suggests that meningiomas in elderly patients grow slower than in younger patients [28], although some long-term data of incidental meningiomas show that the majority of meningiomas eventually grow [18].

For patients with meningioma, surgery is the main treatment modality and offers the possibility of cure [7]. Nevertheless, surgery may cause significant complications [5, 14]. Information about anticipated clinical course, including the short-term risks, is needed for patient information and informed consent. This is a common dilemma in surgery, weighing the risk of short-term complications and neurological impairment against natural history of disease and the expected long-term results. This equation may be especially challenging for asymptomatic patients with presumed WHO grade I meningiomas where the intention of treatment is to delay or prevent later symptoms and possibly provide better chances of a cure, but with no short-term benefit.

It is well known that following brain tumor surgery, transient neurological deficits may occur [11]. Due to the long-term treatment aim in patients with meningiomas (i.e., cure), there are several studies reporting on long-term outcome of patients, but less information is available concerning short-term morbidity [2, 22, 44]. Information on short-term outcome, supplemented by information on expected long-term outcome available in literature, may educate patients and thus facilitate coping in the immediate postoperative period, since the clinical experience indicates that short-term morbidity is far from trivial.

The aim of this registry-based study was to benchmark the complication rate within 30 days in a Swedish nation-wide cohort of meningioma patients using data from the Swedish brain tumor registry (SBTR). Further, we investigated outcomes in the highly relevant group of asymptomatic patients.

## Materials and methods

### The Swedish brain tumor registry

The SBTR is a regionally based nation-wide registry of adult patients (18 years or older) with histopathologically verified brain tumors that was initiated in 1999 [4]. In the Swedish health care system, six different regions provide neurosurgical care to patients with tumors in the central nervous system (CNS). All regions report to SBTR; however, the level of coverage has varied between the different regions over time.

### Study population

We aimed to include all patients in SBTR with intracranial meningioma treated surgically in Sweden from 2009 to 2015 to provide actuality of the current neurosurgical practice. However, in our study, we have only used data from regions where the registration was 80% or more (for any given year) to provide truly representative and population-based data. Registration rate was defined as percentage of diagnoses in the SBTR that corresponds to diagnoses reported to the compulsory National Cancer Registry. For this reason, in one region, we used data from 2012 to 2013 only. In all other regions, the inclusion period for this study was from 2009 through 2015.

### Variables

All regions in Sweden report to the SBTR data concerning baseline characteristics, lead times, and outcomes following treatment. The variables registered in the SBTR following surgery are described in detail in Table 1.

**Table 1:** Definitions of variables

Variable	Definition
Age	Years at time of diagnosis
Sex	Male or female
Symptoms at diagnosis	• Asymptomatic (yes/no) • Focal deficit (yes/no) • Seizure (yes/no) • ICP related (e.g., headache, cognition) (yes/no)
WHO performance status	Grade 0, fully active, able to carry on all pre-disease performance without restriction Grade I, restricted in physically strenuous activity but ambulatory and able to carry out work of a light or sedentary nature, e.g., light house work, office work Grade II, ambulatory and capable of all self-care but unable to carry out any work activities. Up and about more than 50% of waking hours Grade III, capable of only limited self-care, confined to bed or chair more than 50% of waking hours Grade IV, completely disabled. Cannot carry on any self-care. Totally confined to bed or chairGrade V, dead
Date of imaging diagnosis	dd.mm.yyyy
Laterality	Left/right/bilateral
Multifocal	Yes/no
Largest diameter of tumor	< 4 cm 4–6 cm > 6 cm
Date of surgery	dd.mm.yyyy
Type of surgery	Biopsy or resection
Simpson grade	Grade I, total removal Grade II, tumor removal and coagulation of attachment Grade III, tumor removal without coagulation Grade IV, subtotal removal Grade V, decompression/biopsy
Complication within 30 days	Yes/no
New or worsened focal deficit within 30 days	Yes/no
New onset seizure within 30 days	Yes/no
Any infection within 30 days	Yes/no
Any VTE within 30 days	Yes/no
Any hematoma within 30 days	Yes/no
Complication leading to reoperation within 30 days	Yes/no
Date of discharge neurosurgical department	dd.mm.yyyy
Histopathology	*SNOMED codes* Meningioma: 95399, 95301, 95303, 95310, 95330, 95340, 95381, 95383, 95391

### Statistics

All analyses were done with SPSS, version 24.0 (Chicago, IL, USA). Statistical significance level was set to *p* < 0.05 and all tests were two-sided. Central tendencies were presented as means ± SD, or median and interquartile range if skewed. Categorical data were analyzed with Pearson’s Chi-square test, independent sample *t* test, or Mann-Whitney *U* test, as appropriate. For survival, we present Kaplan-Meier curves compared with log-rank test. To identify possible predictors of neurological deficits in asymptomatic patients, we performed a post hoc multivariable logistic regression analysis, selecting predictors based upon presumed clinical relevance.

## Results

### Baseline characteristics

In total, 2324 patients that underwent surgery for intracranial meningioma were included. The mean age was 58.7 years (SD ± 13.5) and 1638 patients were females (70.5%). There were 349 patients (15.0%) with skull base meningiomas. Patients presented most often with focal deficit (*n* = 1450, 62.4%) while 327 patients (14.1%) were asymptomatic. The median time from imaging to surgery was 10 weeks (IQR 4–24). Baseline characteristics are presented in Table 2.

**Table 2:** Baseline characteristics

	Total (*N* = 2324)	Asymptomatic (*N* = 327)	Symptomatic (*N* = 1996)	*p* value
Age, mean (SD)	58.7 (13.5)	56.1 (13.6)	59.1 (13.4)	< 0.001
Age groups				< 0.01
18–39, *n* (%) 40–59, *n* (%) 60–79, *n* (%) 80 or older, *n* (%)	207 (8.9) 924 (39.8) 1084 (46.7) 105 (4.5)	42 (12.9) 140 (42.9) 135 (41.4) 9 (2.8)	165 (8.3) 784 (39.3) 949 (47.6) 96 (4.8)	
Female, *n* (%)	1637 (70.5)	243 (74.3)	1394 (69.8)	0.10
Preop MRI, *n* (%) missing, *n* = 3	2165 (93.2)	292 (90.1)	1873 (93.8)	0.01
Tumor size, *n* (%)				< 0.001
< 4 cm 4–6 cm > 6 cm missing, *n* = 409	1002 (43.1) 660 (28.4) 253 (10.9)	193 (70.2) 58 (21.1) 24 (8.7)	809 (49.3) 602 (36.7) 229 (14.0)	
Skull base, *n* (%)	348 (15.0)	38 (11.6)	310 (15.5)	0.07
Posterior fossa, *n* (%)	215 (5.4)	19 (5.8)	196 (5.3)	0.71
Laterality				0.02
Right, *n* (%) Left, *n* (%) Bilateral, *n* (%) missing, *n* = 377	830 (42.6) 969 (49.8) 147 (7.6)	135 (49.8) 123 (45.4) 13 (4.8)	695 (41.5) 846 (50.5) 134 (8.0)	
Multifocal, *n* (%) missing, *n* = 14	183 (7.9)	35 (10.9)	148 (7.4)	0.04
Asymptomatic, *n* (%) missing, *n* = 1	327 (14.1)	327 (14.1)	–	–
Focal deficit, *n* (%) missing, *n* = 170	1450 (62.4)	–	1450 (62.4)	–
Seizures, *n* (%) missing, *n* = 170	564 (24.3)	–	564 (24.3)	–
ICP related, *n* (%) missing, *n* = 170	863 (40.1)	–	863 (40.1)	–
Performance status, n (%)				< 0.001
0 1 2 3 4 missing, *n* = 108	1041 (47.0) 639 (28.8) 348 (15.7) 163 (7.4) 25 (1.1)	235 (73.2) 58 (18.1) 20 (6.2) 7 (2.2) 1 (0.3)	806 (42.5) 581 (30.7) 328 (17.3) 156 (8.2) 24 (1.3)	
Imaging diagnosis to surgery, median, weeks (IQR)	10 (4–24)	29 (13–93)	8 (3–20)	< 0.001

*p value comparison between symptomatic and asymptomatic

### Clinical outcome

**Fig. 1:**
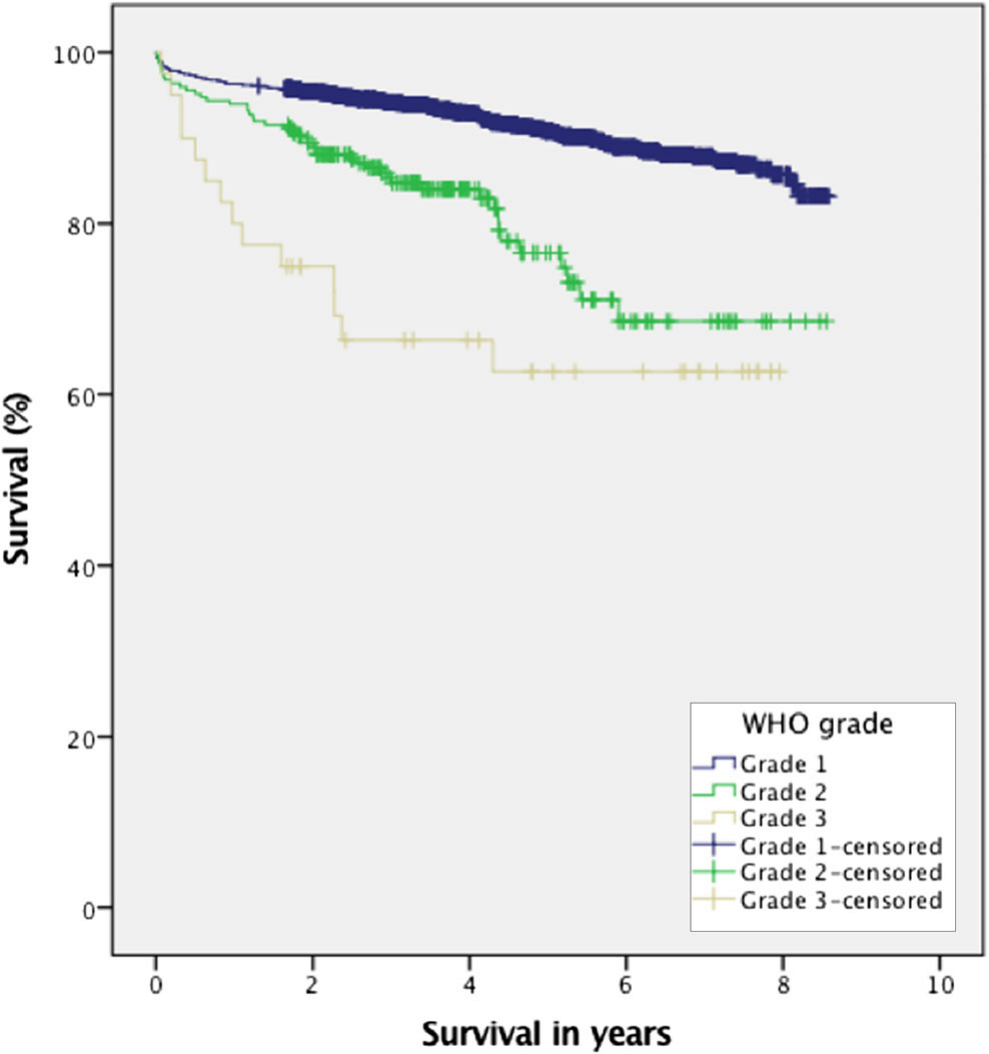
Survival in meningioma patients depending on WHO grade (*p* < 0.001)

Gross total resection (GTR), defined as Simpson grades 1–3, was achieved in 86.6% of the cohort. Focal neurologic deficits occurred in 14.8% of the patients within 30 days of surgery. Venous thromboembolism (VTE) occurred in 3.0% of patients while new-onset seizure occurred in 4.5% of patients. When analyzing patients registered as seizure-free preoperatively, 4.8% experienced new-onset seizures (173 cases without either preoperative or postoperative status excluded from analysis). Symptomatic postoperative hematoma were registered in 9.4% of the patients while reoperation within 30 days due to any complication was performed in 5.2% of the patients. We analyzed if survival differed across WHO groups because we had no central review of pathology, and as shown in Fig. 1, the survival curves separated the groups as expected. Further details are presented in Table 3. The postoperative 30-day mortality was 1.5% in the whole cohort (*p* = 0.06) and 2.4% in patients with higher-grade (WHO grade II and III) meningioma (*p* = 0.14).

**Table 3:** Intraoperative and postoperative variables

	Total (*N* = 2324)	Asymptomatic (*N* = 327)	Symptomatic (*N* = 1996)	*p* value
Simpson grade				< 0.001
Grade I Grade II Grade III Grade IV Grade IV missing = 259	584 (28.3) 1029 (49.8) 175 (8.5) 238 (11.5) 39 (1.9)	101 (36.7) 124 (45.1) 24 (8.7) 17 (6.2) 9 (3.3)	483 (27.0) 904 (50.5) 151 (8.4) 221 (12.4) 30 (1.7)	
New deficit, *n* (%) missing = 4	344 (14.8)	27 (8.3)	317 (15.9)	< 0.001
New seizure, *n* (%) missing = 4	105 (4.5)	12 (3.7)	93 (4.7)	0.43
Hematoma, *n* (%) missing = 3	218 (9.4)	15 (4.6)	203 (10.2)	0.001
Reoperation due to complication, *n* (%) missing = 3	120 (5.2)	7 (2.1)	113 (5.7)	< 0.01
Infection, *n* (%) missing = 4	148 (6.4)	18 (5.5)	130 (6.5)	0.49
VTE, *n* (%) missing = 4	69 (3.0)	2 (0.6)	67 (3.4)	< 0.01
30-day mortality, *n* (%)	34 (1.5)	1 (0.3)	33 (1.7)	0.06
WHO grade 1, *n* (%)	2036 (87.6)	292 (89.3)	1744 (87.4)	0.33
Planned oncological treatment, *n* (%) missing = 32	125 (5.5)	18 (5.6)	107 (5.4)	0.92

### Asymptomatic vs symptomatic patients

**Fig. 2:**
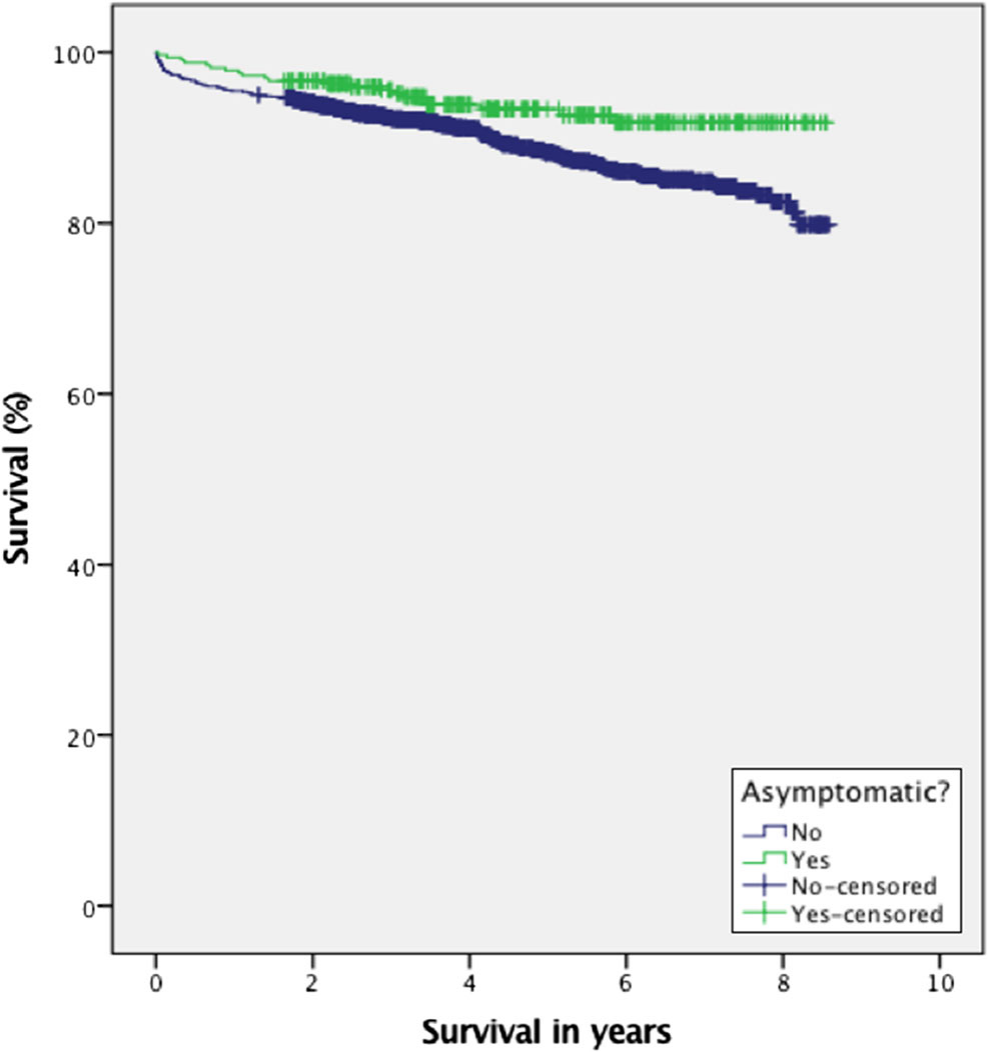
Survival in asymptomatic compared to symptomatic meningioma patients (*p* < 0.01)

Comparison of baseline characteristics and outcomes between symptomatic and asymptomatic patients are provided in Tables 2 and 3. A total of 327 asymptomatic patients (14.1%) underwent surgery for meningioma. The asymptomatic patients ranged from 12.6% in 2014 to 18.2% in 2011, and there was no time trend with increasing numbers of surgery on asymptomatic patients in the observation period (*p* = 0.49). As shown in Table 2, the asymptomatic patients were younger and presented with smaller tumors than the symptomatic group (both *p* < 0.001). Simpson grade 1 and 2 was achieved in 36.7%, respectively, 45.1% in asymptomatic patients. Postoperative mortality within 30 days of surgery was 0.3% in asymptomatic patients compared to 1.7% in the symptomatic patients (*p* = 0.06), with overall survival significantly better in the asymptomatic patients compared with symptomatic patients (*p* < 0.01) (Fig. 2).

### Predictive factors in asymptomatic patients

Due to the significant proportion of postoperative neurological deficits, we explored predictors for new deficits following surgery in asymptomatic patients. Post hoc, we created a multivariable logistic regression model including age, functional status, extension of surgery, sex, meningioma grade, tumor size, and location (skull base and posterior fossa) to identify possible independent predictors for development of new deficit postoperatively. However, we could not identify any predictors based upon the above-mentioned variables.

### Complications in relation to Simpson grade

We also evaluated if complications were associated with Simpson grading. However, no association between Simpson grade and new onset seizure (*p* = 0.42), VTE (*p* = 0.42), postoperative hematoma (*p* = 0.29), postoperative infection (*p* = 0.20), or reoperation due to complications (*p* = 0.63) was observed. Contrary, there was an association between Simpson grade and new or worsened neurological deficit (*p* < 0.001). New or worsened neurological deficit were observed in 14% of Simpson grade 1, 12% of Simpson grade 2, 19% in Simpson grade 3, 25% in Simpson grade 4, and 28% in Simpson grade 5.

## Discussion

In this nationwide registry-based study of patients undergoing surgery for intracranial meningioma, we benchmark the 30-day complication rate for clinically relevant complications. The risk of reoperation within 30 days of surgery due to complications was 5.2%, new focal neurological deficit occurred in 14.8%, new-onset seizures in 4.5%, VTE occurred in 3.0%, and the 30-day mortality was 1.5%. These real-world data on short-term outcomes may be useful in the decision-making process and prior to surgery.

### Perioperative outcomes

We found that the most common postoperative complication to be new onset focal neurological deficit, which is in agreement with previous studies [39]. In the literature, the proportion experiencing new or worsened deficit in unselected patients with meningioma is 8.3–9.3% [9, 39]. A retrospective single-center study from 1984 reported postoperative deficits in 10.8% of patients during the first 30-day postoperative period, and thus, our data with 14.8% new deficits appears unfavorable [9]. However, it is difficult to compare with retrospective assessment with standardized prospective registration due to detection bias [12]. In selected materials, the range is wider and much affected by tumor location and preoperative symptomatology [10, 27, 31]. In general, the literature reports improvement of neurological deficits in most patients during the initial follow-up time after surgery [9, 40]. Additionally, the short-term neurological deficit may also predispose for other medical complications in the postoperative period [39].

Onset of seizures after meningioma surgery is a renowned concern, explaining the interest in prophylactic treatment with antiepileptic drugs (AED) [23], although the current evidence indicates no clear benefits in prevention of postoperative seizures with routine perioperative AED administration [20, 38]. Routine perioperative administration of AED is not performed at any of the neurosurgical centers in Sweden. In this study, new onset seizures occurred in 4.5% and this is in line with the existing literature where new onset seizure postoperatively was reported in the range 1.9 to 19.4% [23, 36, 38, 43]. In a recent systematic review and meta-analysis, among the 1085 patients with supratentorial meningiomas without seizures prior to surgery, new-onset seizures occurred in 12.3% [13]. This large variability between the reported seizure frequency in the literature may be due to different lengths of follow-up, meningioma location, and pattern of evaluation because retrospective cross-sectional studies may capture different aspects than standardized and prospective reporting.

Postoperative hematoma following meningioma surgery was 9.4% and the percentage undergoing reoperation due to any cause within 30 days was 5.2%. The variable “postoperative hematoma” reported in to the SBTR is defined as symptomatic hematoma without additional information regarding what kind of symptoms. Reviewing the literature, postoperative hematomas in need of surgical evacuation were reported in the range of 2.1–7.1% [14, 17, 22]. In a prospective study by Geßler et al. using postoperative imaging in 113 patients with meningioma, there were 30 patients (26.5%) who experienced symptoms postoperatively, including prolonged awakening, seizures, and neurological deficit [15]. A total of 28 patients (24.7%) experienced postoperative symptoms and radiological verified hematoma. Two patients underwent reoperation due to a hematoma, which represents 1.8% of the cohort of 113 patients. The frequency of postoperative hematoma may seem high in our cohort, but since reoperations due to any cause occurred in 5.2% of patients, this seems comparable with previous reports. It is not reported to the registry the cause of operation within 30 days, but according to literature, the majority of the reoperations within 30 days after surgery of intracranial tumors were due to hematomas, leakage of cerebrospinal fluid, or infection [22, 32, 41].

Venous thromboembolism occurred in 3.0% of our cohort, in accordance with previous results where numbers ranged from 3.6 to 7.2% [17, 35]. In SBTR, we are unable to differentiate deep vein thrombosis from pulmonary embolism. The risk-benefit of routine anticoagulation prophylaxis should be carefully weighed given the ratio of hematoma/VTE seen in an unselected meningioma cohort [35]. The timing of complications also indicates that prophylaxis may be safer to delay until > 24 h postoperatively [41].

The 30-day mortality in Sweden after surgery for intracranial meningioma is currently at 1.5%. This corresponds to similar findings in a Norwegian study where the overall surgical mortality of intracranial tumor surgery within 30 days was reported at 2.3% and for only meningiomas 0.9% [22]. As expected, case selection is a strong predictor of outcome because studies on small meningiomas (< 3 cm) and convexity meningiomas show no 30-day mortality [27, 31], and similarly, we present 0.3% mortality within 30 days in asymptomatic patients while 2.4% for patients with higher-grade meningiomas. A study from 1984 showed a mortality rate of 4.0% during the first 30-day postoperative period for intracranial meningiomas [9]; hence, surgical treatment appears safer in modern neurosurgery. This improvement may be due to better surgical and anesthesiologic techniques, but treatment at an earlier time-point with less burden of disease due to better access to imaging may also contribute [37].

### Asymptomatic patients

Due to the usually indolent natural course, some argue that surgery should be reserved for larger meningiomas, meningiomas that exhibit growth, or meningiomas that become symptomatic [8, 16]. Nevertheless, in clinical practice, the treatment plan is often individualized and adjusted for each patient, including patient’s preference. In principle, the treatment should be better than the natural history and, in this regard, a short-term neurological morbidity of 8.3% must be considered. Unfortunately, surgical indications are not reported in this study (e.g., radiological growth, patients wish), and hence, we cannot make direct assumptions of the expected natural course. In the literature, new neurological deficits in the short-term following surgery of asymptomatic patients are reported in a wide range with respect to frequency and severity [21, 29, 44]. Thus, identifying patients with higher risk is of importance and both tumor size and location are presumably important, but also factors such as longer lasting surgery, poor functional status, and high patient age may impact postoperative outcome [5]. We also explored possible predictive factors for neurological deficits after surgery but we were unable to identify any maybe due to rather crude variables.

### Registry-based meningioma studies

Studies relying on data from clinical registries and administrative databases are useful for evaluating treatment strategies and add a different dimension to the results of more selective randomized controlled trials. This kind of research is especially valuable in the field of neurosurgery, where large variations in clinical practice exist [3, 19]. Moreover, clinical registries and administrative databases allow inclusion of large patient groups that are ineligible for inclusion in randomized trials due to age and comorbidity. Additionally, registry-based studies allow monitoring of trends, costs, and complications of surgical procedures in a real-world setting.

Still, registry-based studies must build upon what is reported to a registry, sometimes limiting chances to explore potential interesting associations. However, for variables included, it is possible to collect large amounts of data in a population-based setting. The SBTR collects data from all regions in Sweden and has a good coverage and established systems for quality control, which makes it a useful source of information concerning collected quality metrics. The baseline characteristics captured by SBTR is similar to previous large-scale and population-based reports [1, 39]. Very few studies have been published with registry-based data regarding meningioma using data from the Surveillance, Epidemiology and End Results (SEER) registry [1, 2]. In comparison, the SBTR data contain important information on symptoms and the functional level as shown in Table 1. In addition, the SEER national tumor database collects reports from 20 regional cancer registries, which includes approximately 28% of the population in USA, making this registry not entirely representative of the national meningioma population. Another large registry used for patients with meningioma is the National Cancer Data Base (NCDB) [26]. Two large studies have been performed with data from this registry where the main aim of the studies have been gross total resection and predictors of improved survival as well as factors for survival in meningiomas [25, 34]. These studies do, however, lack the postoperative short-term aspects with regard to complications and surgically acquired deficits, aspects that may be important in the decision-making process.

### Strengths and limitations

Limitations of this study include those inherent to registry-based studies with limited details and without possibility to complete missing data. Specifically, there is a lack of detail for variables including radiological parameters, meningioma location, and radiosurgery. The lack of long-term data if neurological deficits were transient or permanent is another limitation. Also, surgeon-evaluated deficit may not be sensitive for all aspects compared to patient-reported deficit [12]. Finally, some variables may be subject to considerable interpretation (e.g., postoperative hematoma) while others are more robust (e.g., VTE, reoperation due to complication). Strengths include a truly population-based inclusion of a large number of meningioma patients from a recent time period where data are reported prospectively in a continuous and standardized fashion. Due to the regionalized health care system, any major complication in the post-operative course is treated at the same department that performed the primary surgery. Consequently, we expect that most major complications are reported to the SBTR.

## Conclusion

We have in this registry-based study on meningioma benchmarked the 30-day complication rate and, in addition, presented current neurosurgical outcome in relation to preoperative symptoms and WHO grade. Since surgical decision-making is a careful consideration of short-term risk versus long-term benefit, this information may be useful for both caregivers and patients.
